# Extracellular Vesicles Contribute to the Metabolism of Transthyretin Amyloid in Hereditary Transthyretin Amyloidosis

**DOI:** 10.3389/fmolb.2022.839917

**Published:** 2022-03-23

**Authors:** Hiroki Yamaguchi, Hironori Kawahara, Noriyuki Kodera, Ayanori Kumaki, Yasutake Tada, Zixin Tang, Kenji Sakai, Kenjiro Ono, Masahito Yamada, Rikinari Hanayama

**Affiliations:** ^1^ Department of Immunology, Graduate School of Medical Sciences, Kanazawa University, Kanazawa, Japan; ^2^ Department of Neurology and Neurobiology of Aging, Graduate School of Medical Sciences, Kanazawa University, Kanazawa, Japan; ^3^ WPI Nano Life Science Institute (NanoLSI), Kanazawa University, Kanazawa, Japan; ^4^ Department of Internal Medicine, Division of Neurology, Kudanzaka Hospital, Tokyo, Japan

**Keywords:** amyloidosis, transthyretin, extracellular vesicle, ATTRv amyloidosis, atomic force microscope

## Abstract

Hereditary (variant) transthyretin amyloidosis (ATTRv amyloidosis), which is caused by variants in the transthyretin (TTR) gene, leads to TTR amyloid deposits in multiple organs and various symptoms such as limb ataxia, muscle weakness, and cardiac failure. Interaction between amyloid proteins and extracellular vesicles (EVs), which are secreted by various cells, is known to promote the clearance of the proteins, but it is unclear whether EVs are involved in the formation and deposition of TTR amyloid in ATTRv amyloidosis. To clarify the relationship between ATTRv amyloidosis and EVs, serum-derived EVs were analyzed. In this study, we showed that cell-derived EVs are involved in the formation of TTR amyloid deposits on the membrane of small EVs, as well as the deposition of TTR amyloid in cells. Human serum-derived small EVs also altered the degree of aggregation and deposition of TTR. Furthermore, the amount of TTR aggregates in serum-derived small EVs in patients with ATTRv amyloidosis was lower than that in healthy controls. These results indicate that EVs contribute to the metabolism of TTR amyloid, and suggest that TTR in serum-derived small EVs is a potential target for future ATTRv amyloidosis diagnosis and therapy.

## Introduction

Amyloidosis refers to a group of diseases in which insoluble proteins, called amyloids, are deposited in various organs, causing functional impairment (Picken, 2020). Amyloid is formed by the misfolding and aggregation of soluble proteins. Once formed, amyloid takes on a fibrillar structure and is deposited outside the cell (Sipe and Cohen, 2000; Picken, 2020). The extracellular deposition of amyloid leads to destruction of normal tissue structure and cellular dysfunction (Agostinho et al., 2010; Merlini et al., 2011). Physical compression by amyloid deposition, oxidative stress, and abnormal mitochondrial function are thought to be the main mechanisms of cellular dysfunction (Ando et al., 1998; Buxbaum and Linke, 2012). A variety of soluble proteins, including amyloid-β (Aβ), α-synuclein (α-syn), transthyretin (TTR), β2-microglobulin (β2-MG), and serum amyloid A (SAA) are aggregated and deposited in the body, causing amyloidosis (Picken, 2020). For example, Aβ and α-syn are predominantly deposited in the brain, Aβ aggregates are detected in patients with Alzheimer’s disease and are called senile plaques (Hashimoto et al., 2020), and Lewy bodies, formed by the aggregation of α-syn, are deposited in the brains of patients with Parkinson’s disease and dementia with Lewy bodies (Gomperts, 2016). These are examples of localized amyloidosis, in which amyloid deposits are confined to the central nervous system.

In systemic amyloidosis, such as reactive AA amyloidosis and hereditary (variant) transthyretin amyloidosis (ATTRv amyloidosis), amyloid is deposited in multiple organs. Reactive AA amyloidosis, in which SAA is the amyloid precursor protein, is associated with secondary to chronic inflammatory diseases and infections (rheumatoid arthritis, systemic lupus erythematosus, tuberculosis, etc.). In hereditary ATTRv amyloidosis, TTR gene variant forms amyloid, which is deposited in multiple organs such as the peripheral nerves, gastrointestinal tract, heart, eyes, and kidneys (Sekijima et al., 2018). TTR is mainly produced in the liver and exists as a tetramer in serum; however, the dissociation of tetramers into a monomers is the rate-limiting step in amyloid formation (Sekijima et al., 2018). TTR tetramers composed of variant TTR are more likely to dissociate into monomers than those composed of wild-type TTR (Sekijima et al., 2018; Yee et al., 2019). The most common variant in the causative TTR gene is the p.Val50Met (Val30Met; V30M) variant, and the resulting mutated TTR is called V30M-TTR (Yee et al., 2019). Acidic conditions also promote TTR amyloid formation *in vitro* (Yee et al., 2019). Unlike Aβ, which is produced by neurons, TTR is not produced near the site of deposition of TTR aggregation or in the surrounding tissues. Furthermore, the mechanism of TTR aggregation and deposition in the body remains unknown.

Extracellular vesicles (EVs) are small membrane vesicles secreted by various cell and tissue types (Kalluri and LeBleu, 2020). EVs contain biological materials such as proteins, messenger RNA, and microRNA derived from the cells that produce them. Intercellular communication, such as the transport of pathogenic proteins, plays a major role in EVs, and the proteins and RNAs contained in EVs are useful biomarkers of several diseases (Kalluri and LeBleu, 2020). Notably, the addition of Schwann cell-derived EVs or mesenchymal stem cell-derived EVs to mouse models of diabetic neuropathy or mice that were physically subjected to peripheral neuropathy promoted nerve repair (Dong et al., 2019; Fan et al., 2021). *In vitro*, Neuro2a mouse neuroblastoma (N2a) cell-derived EVs (N2a-EVs) promote Aβ aggregation in EVs. In addition, Aβ aggregated on N2a-EVs is easily taken up by microglia and degraded (Yuyama et al., 2012). Furthermore, continuous injection of N2a-EVs into mouse models of Alzheimer’s disease enhanced the phagocytosis of Aβ aggregates by microglia and reduced the amount of amyloid deposition in the brain (Yuyama et al., 2014). Moreover, SAA and SAA oligomers present in plasma EVs also exhibit amyloid-enhancing-factor activity (Tasaki et al., 2010). Human serum EVs (S-EVs) collected by ultracentrifugation contain TTR amyloid (Tong et al., 2017); however, it remains unknown whether EVs contribute to TTR aggregation, deposition, or clearance in hereditary ATTRv amyloidosis. In this study, we show that TTR aggregation is enhanced on EV membranes and that EVs promote cell deposition accompanied by TTR aggregation, even under non-acidic conditions. Furthermore, we observe a decrease in TTR in serum-derived EVs in patients with hereditary ATTRv amyloidosis, indicating that EVs could play an active role in TTR tissue deposition.

## Materials and Methods

### Purification of Serum Extracellular Vesicles

Six patients with ATTRv (V30M-TTR) amyloidosis and six healthy controls provided serum samples in this study. The patients included three males and three females, whereas the healthy individuals were six males. The age of the patients was 56–75, and that of the healthy controls was 22–40. Serum was collected from patients after diagnosis and before the start of treatment for ATTRv amyloidosis. The duration of illness was 3–7 years at the time of blood sampling. All donors provided informed consent, and the study was approved by the Kanzawa University Ethics Committee Reference 2018–238 (3,029).

All serum samples were centrifuged at 3,000 ×g for 10 min and 10,000 × g for 30 min at 4°C, followed by microfiltration with Millipore 0.22 μm filters. Filtrated serum (1 ml) was reacted with MagCapture Exosome Isolation Kit PS (MagCapture) (FUJIFILM Wako, Japan) according to the manual. After the overnight reaction, purified serum EVs were eluted with elution buffer and the EVs were saved (FUJIFILM Wako). Eluted serum EVs were stored at 4°C until further use. The concentration of serum EVs was measured using the Pierce BCA Protein Assay Kit (Thermo Scientific, United States) at 25°C. The number and size distribution of EV particles were measured *via* nanoparticle tracking analysis using a NanoSight LM10 instrument (software NTA 3.1; Malvern Panalytical, United Kingdom) after diluting the EVs 30 times with phosphate-buffered saline (PBS). The particle numbers per frame used were 70 particles/frame for recording of S-EVs in Raw mode.

### Purification of Cell Line-Derived Extracellular Vesicles

HepG2, HEK293T, and NIH3T3 cell lines was purified. Cells (2 × 10^6^) were spread on a 10 cm dish and incubated with Dulbecco’s modified Eagle medium (DMEM) medium (FUJIFILM Wako) containing 10% fetal calf serum (FCS). EVs in the FCS were eliminated by 1,00,000 × g ultracentrifugation. Cell lines were cultured until they reached 90% confluence at 37°C with 5% CO_2_. Then, the culture medium was changed to fresh DMEM containing 2% FCS (2% FCS-DMEM) and incubated for 24 h. The collected conditioned culture media were centrifuged at 3,000 × g for 10 min and 10,000 × g for 30 min at 4°C, followed by microfiltration with Millipore 0.22 μm filters. Filtrated conditioned culture media were reacted with MagCapture, and cell line-derived EVs were eluted using the same procedure as that used for serum EVs. Measurement of collected EV solutions using the Pierce BCA Protein Assay Kit and NanoSight was performed according to the same procedure as that for serum. The particle numbers per frame used were 42, 50, or 39 particles/frame for recording of HepG2, HEK293T, and NIH3T3 cells in Raw mode, respectively.

### Purification of Recombinant Transthyretin

Protein purification was performed as described previously (Kawahara et al., 2008). TTR (NM_000371.4) and V30M were amplified by PrimeSTAR GXL DNA polymerase (Takara, Japan) using primers (5′- ggg​gcc​cct​ggg​atc​TGG​CCC​TAC​GGG​CAC​CGG​T-3′; 5′- gat​gcg​gcc​gct​cga​TCA​TTC​CTT​GGG​ATT​GGT​G-3′) and subcloned pGEX6P1 using In-Fusion HD Cloning Kit (Takara). V30M mutation was generated by using an inverse PCR method (PrimeSTAR GXL) and primers (5′-TGT​GGC​CaT​GCA​TGT​GTT​CAG​AAA​GG-3′; 5′- ACA​TGC​AtG​GCC​ACA​TTG​ATG​GCA​GG-3′). *Escherichia coli* strain BL21 (DE3) pLysS (Biodynamics, DS260) was transfected with the plasmid pGEX6P1-GST-WT-TTR or pGEX6P1-GST-V30M-TTR. After overnight incubation in LB medium at 37°C, the culture medium was transferred into 100–200 ml of LB medium and incubated at 37°C. At the time of OD600 = 0.5, Isopropyl β-D-1-thiogalactopyranoside (IPTG) was added to a final concentration of 0.1 mM and the medium was incubated for 5 h. Cultured *E. coli* was centrifuged, collected, suspended in PBS, and sonicated on ice. Triton-X 100 (Nacalai Tesque, Japan) was added to the final 0.1% and the solutions were centrifuged at 10,000 × g for 30 min at 4°C. The supernatant was then ultracentrifuged at 100,000 ×g for 90 min at 4°C. The supernatant was mixed with Pierce Glutathione Agarose (Thermo Scientific) and rotated overnight at 4°C. Beads were then washed five times and 1 ml of PBS or FCS-free DMEM and 20 μl of Turbo3C protease (FUJIFILM Wako) was added; the mixture was then rotated for 24 h at 4°C. Then, the TTR solution was collected, followed by microfiltration with Millipore 0.22 μm filters. The concentration of filtered purified TTR solution was measured with Pierce 660 nm Protein Assay Reagent (Thermo Scientific) at 25°C, and the TTR solution was stored at 4°C until use.

### Western Blotting Analysis

The SDS-PAGE was performed under reducing condition according to previous study (Tangthavewattana et al., 2019), and immunoblotting was performed as described previously (Kawahara et al., 2011). Samples were boiled with sodium dodecyl sulfate sample buffer, denatured on 13% polyacrylamide gels, transferred onto a nitrocellulose membrane, and blocked in 4% skim milk in TBS-T (0.5% Triton-X 100 with TBS) for 30 min at room temperature then washed with TBS-T three times. The membrane was then incubated with primary antibodies overnight at 4°C. The membrane was washed three times with TBS-T and incubated with secondary antibodies overnight at 4°C. Both the primary and secondary antibodies were diluted 1,000 times with Can Get Signal 1 and Can Get Signal 2 (TOYOBO, Japan). Following TBS-T washing in triplicate, the membrane was enclosed with SuperSignal West Pico PLUS Chemiluminescent Substrate (Thermo Scientific) or ImmunoStar LD (FUJIFILM Wako). Chemiluminescence was detected using FUSION SYSTEM (Vilber Lourmat, France). The signal intensity of the bands was measured using Evolution Capt Version 18.04 (Vilber Lourmat). The primary antibodies used in this study were anti-prealbumin antibody (Abcam, ab-92469), Anti-CD9, monoclonal antibody (1 K) (FUJIFILM Wako, 014-27763), LEAF Purified anti-human CD63 antibody (BioLegend, 353014), anti-Alix antibody (Cell Signaling Technology, 2171), Anti-HSP70 antibody (BioLegend, 648002), Anti-CD81 antibody (BioLegend, 104902), and Purified Mouse Anti-Flotillin-2 antibody (BD Biosciences, 610384). The secondary antibodies were anti-rabbit IgG, HRP-linked antibody (Jackson Immuno Research Laboratories, 711-035-152), and anti-mouse IgG, HRP-linked antibody (Cell Signaling Technology, 7076P2).

### Thioflavin T Assay

The acidic buffer was 50 mM sodium acetate and 100 mM NaCl at pH 4.2, as described previously (Saito et al., 2005). Cell line-derived EVs (50 μg/ml) and purified TTR in PBS (WT-TTR or V30M-TTR, 0.75 mg/ml) were mixed with the acidic buffer in a PCR tube. The final concentration of TTR was 0.1 mg/ml. The sample solutions were incubated at 37°C in a thermal cycler. The increase in aggregated TTR was evaluated by measuring the fluorescence intensity (FI) of ThT. The ThT solution (as described previously in Saito et al. (2005) (5 μM ThT and 50 mM glycine-NaOH buffer, pH 9.0) and sample solutions were mixed at a ratio of 20:1. The mixture was poured into four wells of a Nunc Black polystyrene 96-well microplate (Thermo Scientific) with 200 μl per well. The FI value was recorded at 25°C within a few minutes of pouring. The measurement device was an Enspire Multimode Plate Reader (PerkinElmer, United States). The measurement timing was as follows: 0 h (immediately after mixing), 6, 12, 24, and 48 h. The wavelengths of the plate reader for excitation and emission were 450 and 482 nm, respectively.

### Biotinylation of Anti-Transthyretin Antibody

EZ-Link NHS-LC-LC-Biotin (Thermo Scientific) was diluted in 2 mM using dimethyl sulfoxide, and 6.7 μl of the diluted biotin solution was mixed with 200 μl of purified anti-transthyretin aggregated antibody (BioLegend, 848202). The mixture was then rotated at 4°C overnight. Then, the same amount of 100 mM glycine-PBS was added to inactivate the remaining biotin.

### Enzyme-Linked Immunosorbent Assay of Extracellular Vesicles (EV ELISA)

A PS Capture Exosome ELISA Kit (Streptavidin HRP) (FUJIFILM Wako, Japan) was used for semi-quantification of EVs. Cell line-derived EVs or serum were used as the samples. The samples were diluted to the desired ratio using the reaction buffer in the kit. Diluted samples were poured into three wells of the kit (100 μl per well) and incubated overnight with shaking at 4°C and 500 rpm. Subsequent procedures were performed according to the manufacturer’s instructions. Control biotinylated anti-CD63 antibody in the kit or biotinylated anti-aggregated TTR antibody described in the previous section were used for the detection of proteins contained in the EVs. Absorbance spectra were obtained at 25°C using an Enspire Multimode plate reader (PerkinElmer), and the absorbance was measured at 450 nm (reference wavelength: 620 nm). According to the manual, the actual absorbance was obtained by subtracting the blank absorbance from the average absorbance at three points.

### Binding of EVs and Purified TTR in Neutral Conditions and Extracellular Vesicles Labeling

Each 30 μl of purified TTR in FCS-free DMEM (WT-TTR or V30M-TTR, 0.4 mg/ml) and cell line-derived EVs (40 μg/ml) were mixed in a PCR tube. The mixtures were incubated for 48 h at 37°C. Then, 15 μL of each sample was used for western blotting analysis, and the remaining samples were diluted 100 times with reaction buffer and used for EV ELISA. The experiment was also conducted in serum EVs using the same procedure. For PKH-labeled EVs, 293T-EVs (3 μg) was incubated with 4x PKH26 Dye solution (Sigma-Aldrich) for 15 min at room temperature according to the manual after both ultracentrifugation followed by MagCapture. PKH-labeled EVs was purified by Exosome Spin Columns (Thermo) by centrifuging at 750 × *g* for 2 min.

### Immunocytochemistry

Immunocytochemistry was performed as described previously (Kawahara et al., 2008). V30M-TTR in FCS-free DMEM was aggregated in acidic buffer for 3 days, and TTR aggregates were diluted in FCS-free DMEM (final TTR concentration; 1.0 μg/ml). HEK293T cell was incubated with 10% FCS-DMEM (500 μl) on gelatin coated 12 mm coverslips until they reached 50% confluence at 37°C. Then, the culture medium was replaced with fresh 2% FCS-DMEM (500 μl) and 10 μl of diluted aggregated TTR was added, and the cells were incubated at 37°C for 30 h. After incubation, the cells were washed with TBS-T and fixed with 4% PFA for 15 min at room temperature. The cells were then washed, 2% bovine serum albumin (BSA) was added to TBS-T, and cells were incubated with shaking for 30 min at room temperature. Then, the cells were washed with TBS-T, and biotinylated anti-aggregated TTR antibody was diluted 1,000 times with 1% BSA in TBS-T and incubated overnight at 4°C. The cells were washed with TBS-T, Streptavidin-Cy3 (BioLegend, 405215) diluted 1,000 times with 2% BSA in TBS-T, and incubated overnight at 4°C with shading. After incubation, the cells were washed with TBS-T and incubated with VECTASHIELD Mounting Medium with DAPI (5 μg/ml) (VECTOR LABORATORIES, Unites states). Purified V30M-TTR in FCS-free DMEM was also used. HEK293T cell was cultured as described above until they reached 90% confluence. The culture medium was then replaced with fresh 2.5% FCS-DMEM (400 μl). Purified V30M-TTR in FCS-free DMEM (50 μl, 2.0 mg/ml) with 50 μl of serum EVs (70 ng/ml) or elution buffer (MagCapture, FUJIFILM Wako, Japan) were also spread on the cell lines, and cells were incubated at 37°C for 18 h. After incubation, the same procedure described above was performed to observe the fluorescence. For the uptake of 293T-EVs, HEK293T cell was cultured as described above until they reached 90% confluence. The culture medium was then replaced with fresh 2.5% FCS-DMEM (400 μl). Purified TTR or V30M-TTR in FCS-free DMEM (50 μl, 2.0 mg/ml) with 50 μl of PKH26-labeled 293T-EVs (70 ng/ml) or PKH26-labeled 293T-EVs alone were also spread on the cell lines, and cells were incubated at 37°C for 24 h. After incubation, the same procedure described above was performed to observe the fluorescence using anti-aggregated TTR antibody, anti-mouse IgG, Biotin-linked antibody (Jackson Immuno Research Laboratories, 715-065-150), and Streptavidin-FITC (BD Bioscience, 554060). They were captured by BZ-X710 (KEYENCE, Japan) or FV10-ASW (Olympus) and Quantitative analysis were performed using Hybrid Cell Count software according to the manual (Keyenece, BZ-H3C).

### High-Speed Atomic Force Microscopy

A laboratory-built high-speed atomic force microscope (HS-AFM) was used for the analysis, as described previously (Ando et al., 2013). In brief, a glass sample stage (diameter, 2 mm; height, 2 mm) with a thin mica disc (1.5 mm diameter and ∼0.05 mm thickness) glued to the top by epoxy was attached onto the top of a Z-scanner using a drop of nail polish. A drop (2 μl) of the serum EV solution from the healthy control group (1.8 × 10^9^ particles/ml) was deposited onto a freshly cleaved mica surface and left to sit for 3 min. This ensured that the S-EVs were nonspecifically immobilized on the mica surface. After rinsing the surface with either PBS buffer or acidic buffer (pH 4.0) of 20 μl, the sample stage was immersed in a liquid cell filled with either PBS buffer or acidic buffer (60 μl), and HS-AFM imaging was conducted in tapping mode. We used small cantilevers (BL-AC10DS-A2, Olympus, Tokyo) with a spring constant, resonant frequency in water, and quality factor in water of ∼0.1 N/m, ∼0.5 MHz, and ∼1.5, respectively. The probe tip was grown on the original tip end of the cantilever through electron beam deposition using ferrocene. The cantilever’s free oscillation amplitude *A*_0_ and set-point amplitude *A*_s_ were set to ∼2 nm and ∼0.9 × *A*_0_, respectively. In some experiments, purified V30M-TTR solution (5 μL) was injected into the observation buffer during HS-AFM imaging so that the final concentration of TTR was either 0.07 mg/ml or 0.25 mg/ml, as described previously (Nonaka et al., 2020). Details of the HS-AFM imaging method are described elsewhere (Uchihashi et al., 2012). Data analysis of HS-AFM images was performed as described previously (Kori et al., 2019; Kodera et al., 2021).

### Analysis of High-Speed Atomic Force Microscopy Images

For analysis, HS-AFM images were pretreated with a low-pass filter to remove spike noise and with a flatten filter to render the overall xy-plane flat using a laboratory-built software, as described before (Ngo et al., 2015). The heights of S-EVs were measured semi-automatically using the following steps. First, the estimated highest point of a S-EV was selected manually. Second, the actual highest point was automatically determined by searching a 10 × 10 pixel area (typically 40 × 40 nm^2^) around the selected point.

2D correlation coefficients were calculated between the HS-AFM images of the first frame and each of the frames within the region of interest (ROI) (i.e., the first frame is the reference) (Kori et al., 2019). The ROIs were set as rectangles that enclosed the spherical-shaped S-EVs (typically 50 × 50 nm^2^). The 2D correlation coefficient was calculated frame-by-frame for each ROI. The 2D correlation coefficient *r* is defined as,

$$r=\frac{\sum_{m}\sum_{n}\left(H_{mn}-\bar{H}\right)\left(R_{mn}-\bar{R}\right)}{\sqrt{\left(\sum_{m}\sum_{n}{\left(H_{mn}-\bar{H}\right)}^{2}\right)\left(\sum_{m}\sum_{n}{\left(R_{mn}-\bar{R}\right)}^{2}\right)}}$$

in which *H*_mn_ and *R*_mn_ are the heights at the pixel points (m, n) in the ROI to be analyzed and the ROI of the reference, respectively.

### Statistical Analysis

Statistical analysis was performed using GraphPad Prism 8.4.3. Statistical comparisons were performed as indicated in the figure legends. *p* values ≤0.05 were considered to be statistically significant, and those not significant (n.s.) were indicated accordingly. Bar graphs are presented as the mean ± standard error (S.E.). Two-sample tests were performed by Mann–Whitney’s U-test. Multiple comparisons were performed via one-way analysis of variance. No statistical methods were used to select *N* number.

## Results

### Extracellular Vesicles Bind to Transthyretin and Promote Transthyretin Aggregation

**FIGURE 1:**
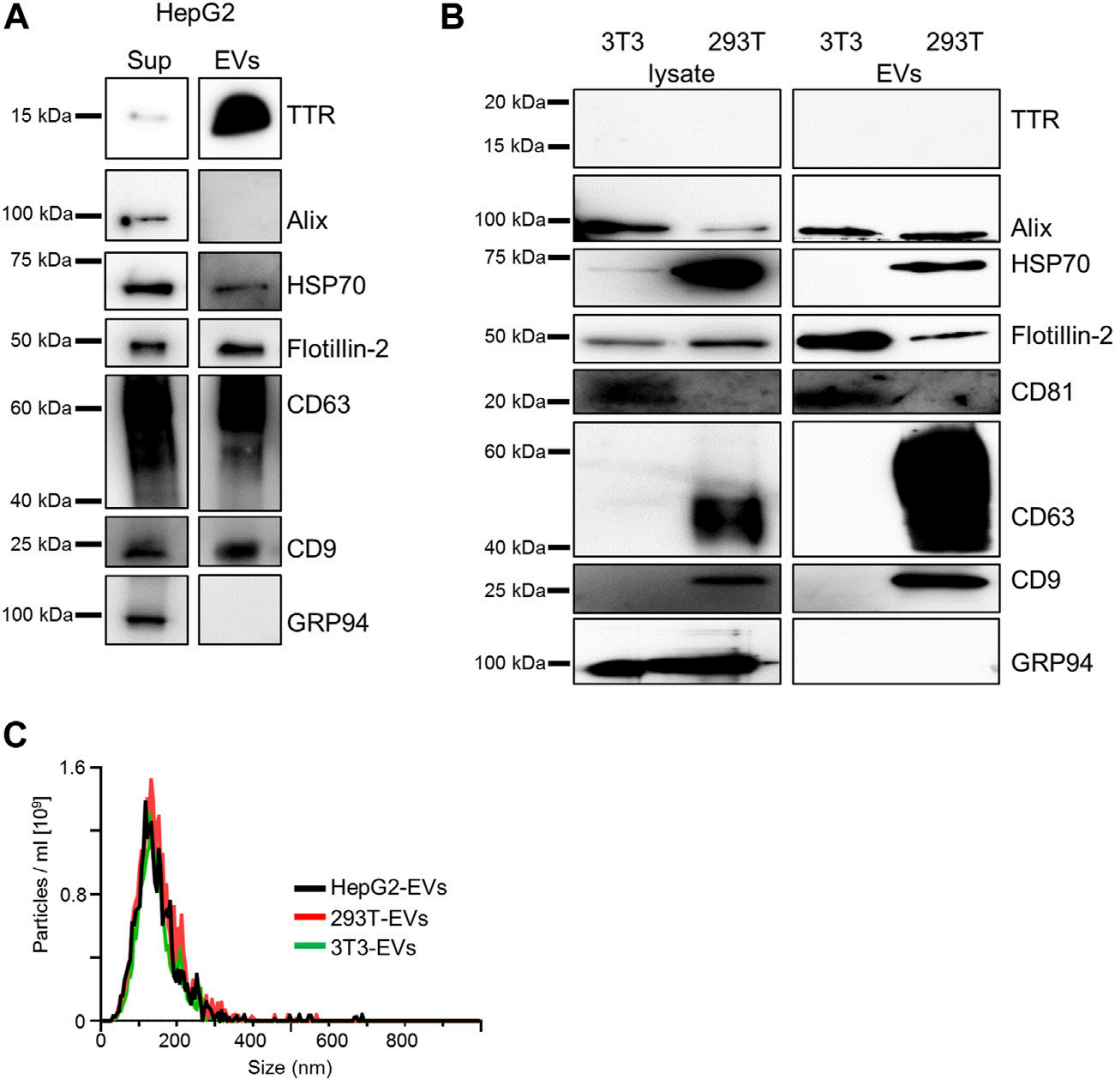
HepG2-derived EVs contain TTR whereas HEK293T-derived EVs or NIH3T3-derived EVs do not. **(A)** TTR monomer was detected in western blotting analysis of the culture supernatant of HepG2 and HepG2-derived EVs. **(B)** TTR was not detected in western blotting analysis of the cell lysate and cell line-derived EVs in HEK293T and NIH3T3. 3T3 (NIH3T3), 293T (HEK293T). **(C)** Analysis of three cell line-derived EVs [HepG2 (black), HEK293T (red), and NIH3T3 (green)] by NanoSight (Raw mode). The diameter and distribution of cell line-derived EVs were similar among the cell lines. All EVs were 40 ng/ml before measurement and were diluted 30-fold with PBS immediately before analysis. Particle concentrations were as follows: HepG2 cell-derived (2.52 × 10^10^ ± 1.19 × 10^9^), HEK293T-derived (2.96 × 10^10^ ± 1.01 × 10^9^), and NIH3T3-derived EVs (2.23× 10^10^ ± 1.28 × 10^9^).

**FIGURE 2:**
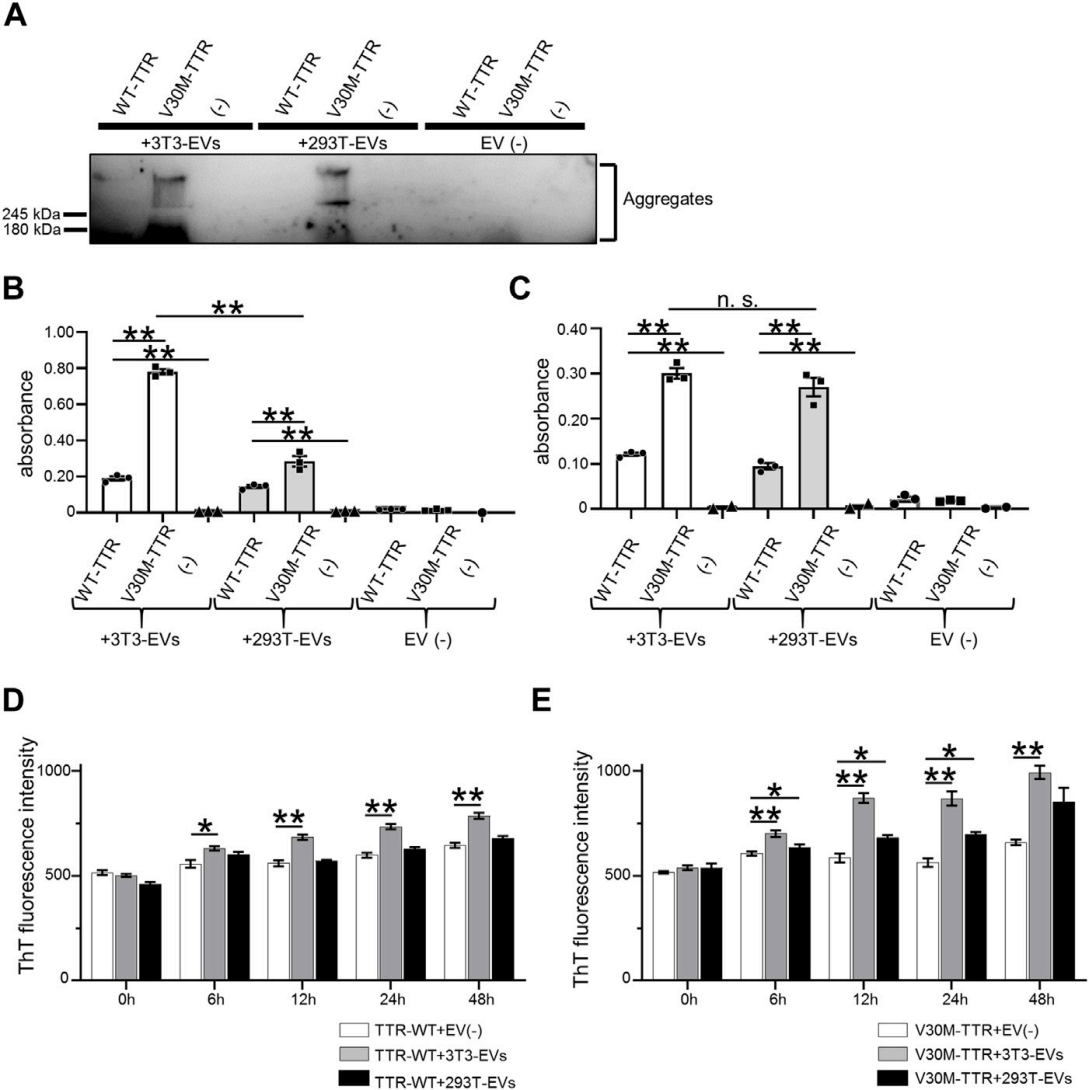
EVs are easily combined with purified V30M-TTR. **(A)** Mixture of NIH3T3-derived EVs, HEK293T-derived EVs or non-EVs, and purified TTR in non-shaking conditions for 48 h used for western blotting analysis. Ladder bands suggestive of aggregates were confirmed above the top of the running gel on V30-TTR and both EVs. **(B)** Remaining samples were diluted and used for EV ELISA. Binding of cell line-derived EVs to TTR was indicated by the increased absorbance in EV ELISA. NIH3T3-derived EVs were more likely to bind to TTR than HEK293T-derived EVs. N = 3, mean ± S.E.; **, *p* < 0.005; n.s., not significant, ANOVA with Tukey’s post hoc test. **(C)** The same samples as the previous experiment were shaken at 500 rpm and used for EV ELISA. TTR-V30M with cell line-derived EVs showed higher absorbance than TTR-WT. N = 3, mean ± S.E.; **, *p* < 0.005; n.s., not significant, ANOVA with Tukey’s post hoc test. **(D)** Increase of Thioflavin T (ThT) fluorescence intensity (FI) in a mixture of EVs and purified WT-TTR with acidic buffer observed by ThT assay. FI value decreased in the following order: TTR with NIH3T3-derived EVs, HEK293T-derived EVs, and elution buffer in both WT-TTR and V30M-TTR. N = 4, mean ± S.E.; *, *p* < 0.05; **, *p* < 0.005; ANOVA with Tukey’s post hoc test. **(E)** Same experiment with V30M-TTR. FI value decreased in the same order as for WT-TTR. FI value of V30M-TTR was generally higher than that of WT-TTR. N = 4, mean ± S.E.; *, *p* < 0.05; **, *p* < 0.005; ANOVA with Tukey’s post hoc test.

The majority of TTR is produced as a secreted protein in the liver (Sekijima et al., 2018); the presence of serum EVs containing TTR has also been reported (Tong et al., 2017). To confirm whether HepG2 cells contained TTR, immunoblotting was performed using HepG2 culture supernatants. TTR was detected in HepG2-EVs (Figure 1A). In HEK293T and NIH3T3 cells that did not produce TTR, TTR was not detected in either the supernatants or EVs (Figure 1B). The particle size and distribution of EVs were similar among the three groups (Figure 1C). Aβ, which is a major causative factor of Alzheimer’s disease, binds to the surface proteins of Neuro2a cell-derived EVs and promotes Aβ aggregation on EVs (Yuyama et al., 2012). To investigate whether TTR, a major causative factor of ATTR amyloidosis (which is the same type of amyloidosis as Alzheimer’s disease), interacts with the EV membrane surface, we analyzed the interaction using purified TTR and EVs derived from HEK293T and NIH3T3 cells that do not produce TTR. HEK293T cell-derived EVs (HEK293T-EVs) or NIN3T3 cell-derived EVs (NIH3T3-EVs) were mixed with purified wild-type TTR (WT-TTR) or V30M-TTR then analyzed. First, immunoblotting was performed using samples that had been mixed with EVs or non-EVs and purified TTR or FCS-free DMEM, then allowed to stand at 37°C for 48 h. In the mixed sample of V30M-TTR and EVs, multiple bands were detected (Figure 2A). This result suggests that mixing of V30M-TTR and EVs resulted in the aggregation of TTR (Figure 2A). The mixed sample of NIH3T3-EVs and WT-TTR also showed slight banding. Multiple bands were not observed in the sample without EVs (Figure 2A). Next, to confirm the co-localization of EVs with TTR aggregates, we performed EV ELISA using the same samples as for immunoblotting. An increase in absorbance was observed in the mixed samples of EVs and TTR, with the highest absorbance in the mixed samples of NIH3T3-EVs and V30M-TTR (Figures 2B,C). The absorbance exhibited a minimal increase in the mixed samples of TTR and non-EVs. Under neutral conditions, EVs from TTR-non-producing cell lines bound to purified TTR and promoted TTR aggregation. In addition, NIH3T3-EVs showed a higher affinity for TTR than HEK293T-EVs.

TTR aggregation is promoted under acidic conditions (Colon and Kelly, 1992). EV-affected TTR aggregation was analyzed using the thioflavin T (ThT) assay. EVs or non-EVs, purified WT-TTR or V30M-TTR, and acidic buffer were mixed and evaluated (Figures 2D,E). WT-TTR showed a significant increase in FI values in the presence of NIH3T3-derived EVs. V30M-TTR showed an increasing trend in FI values in the presence of both NIH3T3-derived and 293T-derived EVs, with 293T-derived EVs showing a significant increase in FI values at 12 and 24 h. Overall, the FI values of V30M-TTR were higher than those of WT-TTR. NIH3T3-EVs and V30M-TTR bound easily, even under acidic conditions, indicating that NIH3T3-EVs are likely to promote the aggregation of TTR.

### Serum Extracellular Vesicles Contain Transthyretin and Transthyretin Aggregates, and Serum Extracellular Vesicles Bind to Purified Transthyretin

**FIGURE 3:**
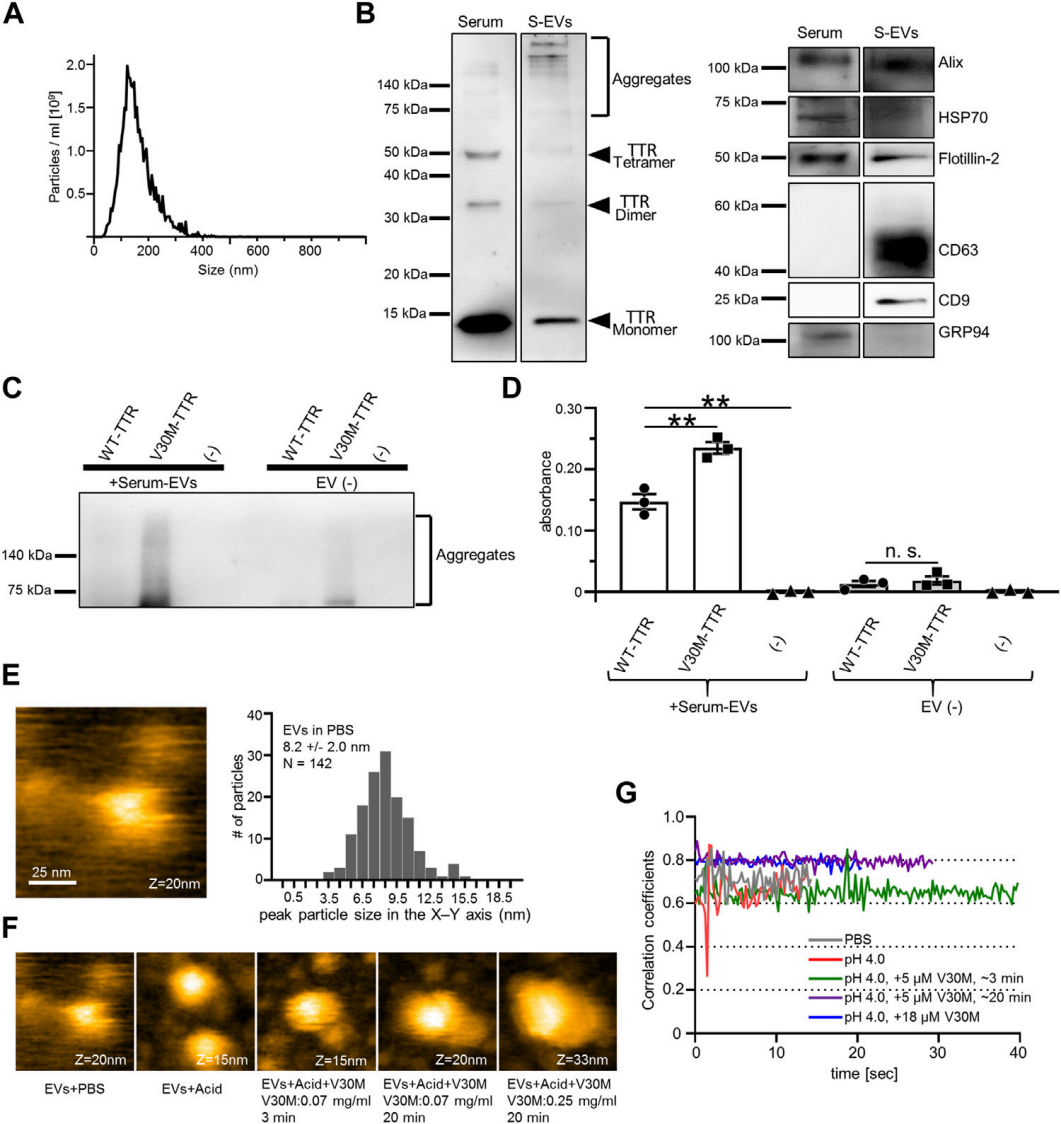
EVs derived from human serum contain TTR, and purified TTR binds to the serum EVs. **(A)** Analysis of EVs derived from serum (S-EVs) by NanoSight (Raw mode). The particle concentration was 4.15 × 10^10^ ± 3.30 × 10^9^. **(B)** Multiple molecular weight TTR and TTR aggregates were detected in western blotting analysis of serum and S-EVs. **(C)** Mixture of S-EVs or Elution Buffer and purified TTR in static conditions for 48 h used for western blotting analysis. V30M-TTR with or without S-EVs exhibited ladder bands. **(D)** Remaining samples were used for EV ELISA. S-EVs with V30M-TTR showed the highest absorbance. S-EVs with WT-TTR also exhibited an increase in absorbance, whereas TTR without EVs showed little increase in absorbance. N = 3, mean ± S.E.; **, *p* < 0.005; n.s., not significant, ANOVA with Tukey’s post hoc test. **(E)** S-EVs in PBS were imaged with HS-AFM. EV particles were observed fixed to the substrate. Particles were mainly smaller than those confirmed by Nanosight. **(F)** Change in S-EV particle height over time under five conditions. Height of serum EVs increased in a time-dependent and TTR-concentration-dependent manner. Acid buffer (Acid). All AFM movies were taken at 250 ms/frame. Scanning area was 100 × 100 nm^2^ with 80 × 80 pixels. **(G)** Time course of 2D correlation coefficients of the surface of EVs under the above five conditions. The 2D correlation coefficient calculation has been described in the Materials and Methods “Analysis of HS-AFM Images” section in detail. The addition of TTR and an increase in TTR concentration reduced amplitude of the EV surface.

Reportedly, S-EVs recovered by ultracentrifugation contain TTR aggregates (Tong et al., 2017). However, these S-EVs also contain many impurities. Some methods have been used to purify S-EVs, including a two-step purification method using size exclusion chromatography in combination with the density gradient method (Onódi et al., 2018). We have reported that EVs can be easily purified by the MagCapture method to ensure their high purity (Nakai et al., 2016). To investigate whether highly purified S-EVs also contain TTR and TTR aggregates, S-EVs from healthy individuals were collected by MagCapture and analyzed using the same procedure as that used for cell line-derived EVs. The particle size of S-EVs was analyzed using a nanoparticle meter, which revealed a similar particle size and distribution to those of cell line-derived EVs (Figures 1C, 3A). TTR with multiple molecular weights was detected in the immunoblot of S-EVs, as well as a small amount of TTR aggregates in the high purity S-EV (Figure 3B). As cultured cell-derived EVs promoted TTR aggregation, we performed a similar analysis with serum-derived EVs. S-EVs or non-EVs were mixed with purified TTR or TTR minus as controls. A ladder-like band was observed in the mixed sample of S-EVs and V30M-TTR, indicating that S-EVs promoted the aggregation of TTR (Figure 3C). In addition, to investigate whether TTR aggregation occurred on the EV surface, EV ELISA was performed using the same samples as those used for immunoblotting. The absorbance increased in the mixed sample of TTR and S-EVs and was higher in V30M-TTR than in WT-TTR (Figure 3D). However, the absorbance did not increase as much as when the cell line-derived EVs were mixed with purified TTR (Figures 2B, 3D). These ELISA results indicated that V30M-TTR was more easily aggregated on the surface of the S-EVs.

As it is difficult to distinguish between S-EVs and lipoprotein impurities by Nanoparticle Tracking Analysis (NTA), it is recommended to combine NTA with microscopic analysis (Bachurski et al., 2019). Hence, HS-AFM was used to visually analyze the binding of S-EVs to purified TTR. S-EVs in PBS were fixed to the substrate and were flexibly fluctuated due to their presence in PBS (Supplemental Figure S1). S-EVs with a height of approximately 20 nm in the Z-axis were mainly observed by HS-AFM: The difference in the height of the S-EVs between Nanosight and HS-AFM was thought to be due to the fact that smaller particles diffuse faster and are preferentially anchored to the substrate, and that the core size of particles was small enough to be observed by HS-AFM, but was effectively estimated larger by the existence of molecules outside S-EVs in Nanosight (Figure 3E). There have been reports of similar results, including one from our group (Woo et al., 2016; Lim et al., 2020, 2021). Furthermore, the detection limit of NTA is about 60 nm (Bachurski et al., 2019), and HS-AFM can detect smaller S-EVs that cannot be detected by NTA. The peak particle size in the X–Y axis was 8.2 nm (Figure 3E). The binding of V30M-TTR to S-EVs was analyzed under acidic conditions. The height of the S-EVs immobilized on the substrate increased in a time-dependent manner and V30M-TTR-concentration-dependent manner (Figure 3F). As the increase in the size of the EVs surface has resulted in a decrease in mobility and amplitude, the fluctuations were suppressed when S-EVs and V30M-TTR interacted with correlation coefficients: Notably, the surface mobility of EVs appeared to reduce upon addition of TTR. This was confirmed by the correlation coefficient analysis for the shapes of S-EVs. Thus, the increase in the height of the S-EVs has resulted in a decrease in the surface mobility of S-EVs (Figure 3G). These data suggest that purified TTR binds to S-EVs and accumulated on EV membranes.

### Serum-Derived Extracellular Vesicles are Involved in Transthyretin Aggregation and the Deposition of Transthyretin Aggregates on Cells

**FIGURE 4:**
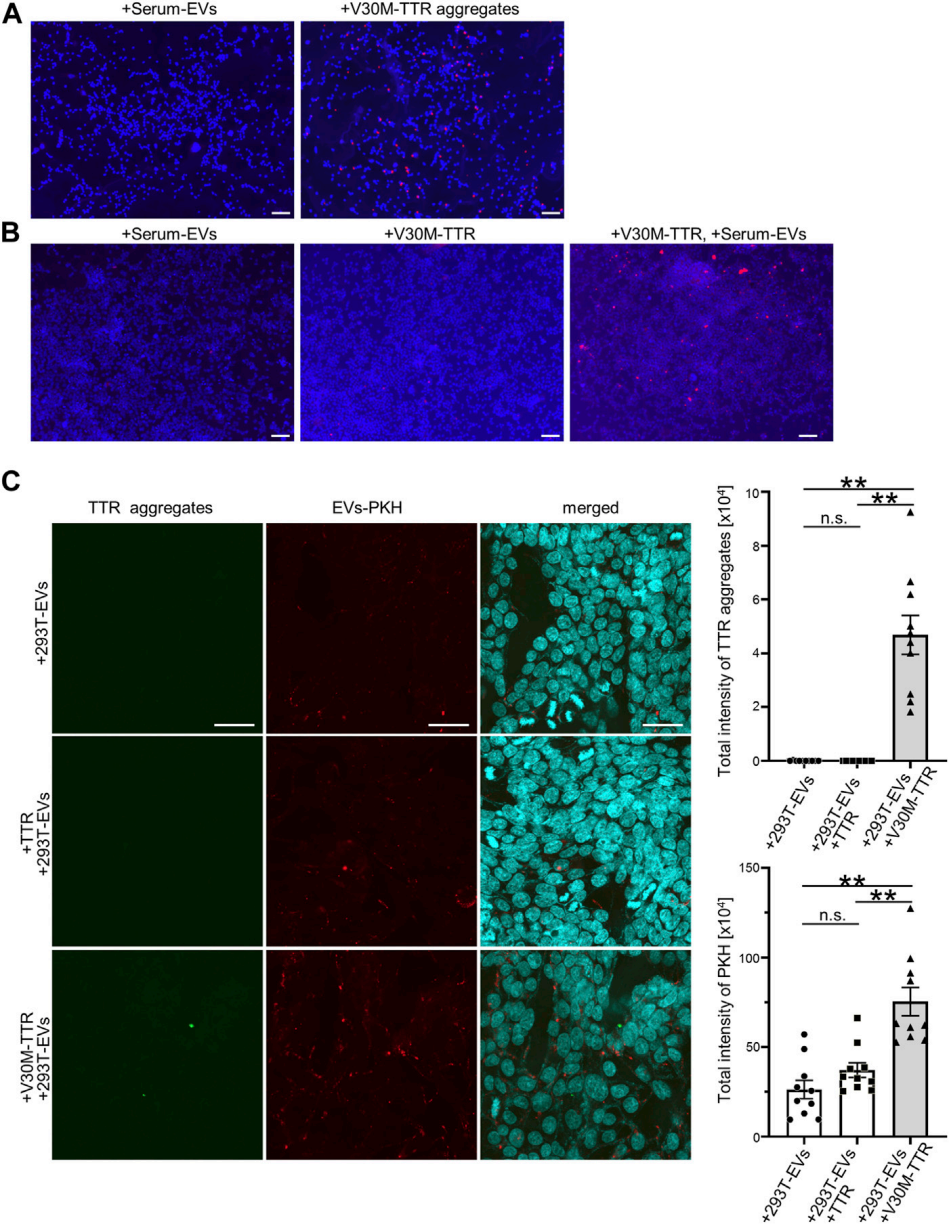
EVs promote the cell deposition of V30M-TTR, and S-EVs change the degree of TTR aggregation and deposition of TTR aggregates on cell lines. **(A)** TTR aggregates spread on HEK293T. Deposited TTR aggregates were detected by the biotinylated TTR aggregate antibody as red fluorescence in immunocytochemistry. Bars: 100 μm. **(B)** Purified V30M-TTR monomer, V30M-TTR monomer with S-EVs, and S-EVs spread on HEK293T. HEK293T showed red fluorescence in only V30M-TTR with S-EVs in appearance. Bars: 100 μm. **(C)** Purified TTR monomer with 293T-EVs, V30M-TTR monomer with 293T-EVs, and 293T-EVs spread on HEK293T. PKH26-labeled 293T-EVs and purified TTR protein (WT or V30M) were mixed and incubated with 293T cells at 37°C for 24 h. The uptake efficiency of EVs was quantified by PKH (right panel), and TTR aggregates were quantified using anti-aggregated TTR antibody (right panel). TTR aggregates were observed only in the presence of V30M-TTR and EVs under this condition. HEK293T showed green fluorescence in only V30M-TTR with 293T-EVs in appearance. Red fluorescence is indicated PKH-labeled EVs that is purified by ultracentrifugation and Magcapture followed by Exosome Spin Columns. Bars: 40 μm. N = 10, mean ± S.E.; **, *p* < 0.001; n.s., not significant, ANOVA with Tukey’s post hoc test.

TTR aggregates are deposited on the outside of cells in the stroma. To investigate whether V30M-TTR aggregates were capable of being deposited on the cell periphery, V30M-TTR aggregates, purified V30M-TTR, and S-EVs were added to the culture cells. First, to confirm the pattern of extracellularly deposited V30M-TTR aggregates, a certain amount of V30M-TTR aggregates was added to HEK293T cells. As a result of analysis using TTR aggregate antibodies, almost no deposition signal was detected in cells incubated with S-EVs purified by MagCaputre compared to those incubated with V30M-TTR aggregates (Figure 4A). Next, we examined whether TTR was aggregated and deposited around the cells when purified V30M-TTR was added to the cell lines. Specifically, 0.22 μm of filtered purified V30M-TTR was added to HEK293T cells, and TTR aggregates deposited around the cells were detected by the same method, as shown in Figure 4A. V30M-TTR aggregates were not detected in V30M-TTR alone to the same extent as in S-EVs (Figure 4A). However, when V30M-TTR and S-EVs were simultaneously added, cellular deposition of TTR aggregates was more pronounced than that under other conditions (Figure 4B). As PKH-labeled EVs is also likely to observe impurities other than EVs (Takov et al., 2017), the analysis was performed using 293T-EVs purified and labeled by spin column after ultracentrifugation followed by MagCapture. V30M with 293T-EVs not only observed the presence of TTR aggregates but also increased EVs uptake (Figure 4C). These data indicate that S-EVs promote cell deposition with aggregation of V30M-TTR.

### Amount of EV Markers and TTR Aggregates in Serum EVs Differ Between Patients and Controls

**FIGURE 5:**
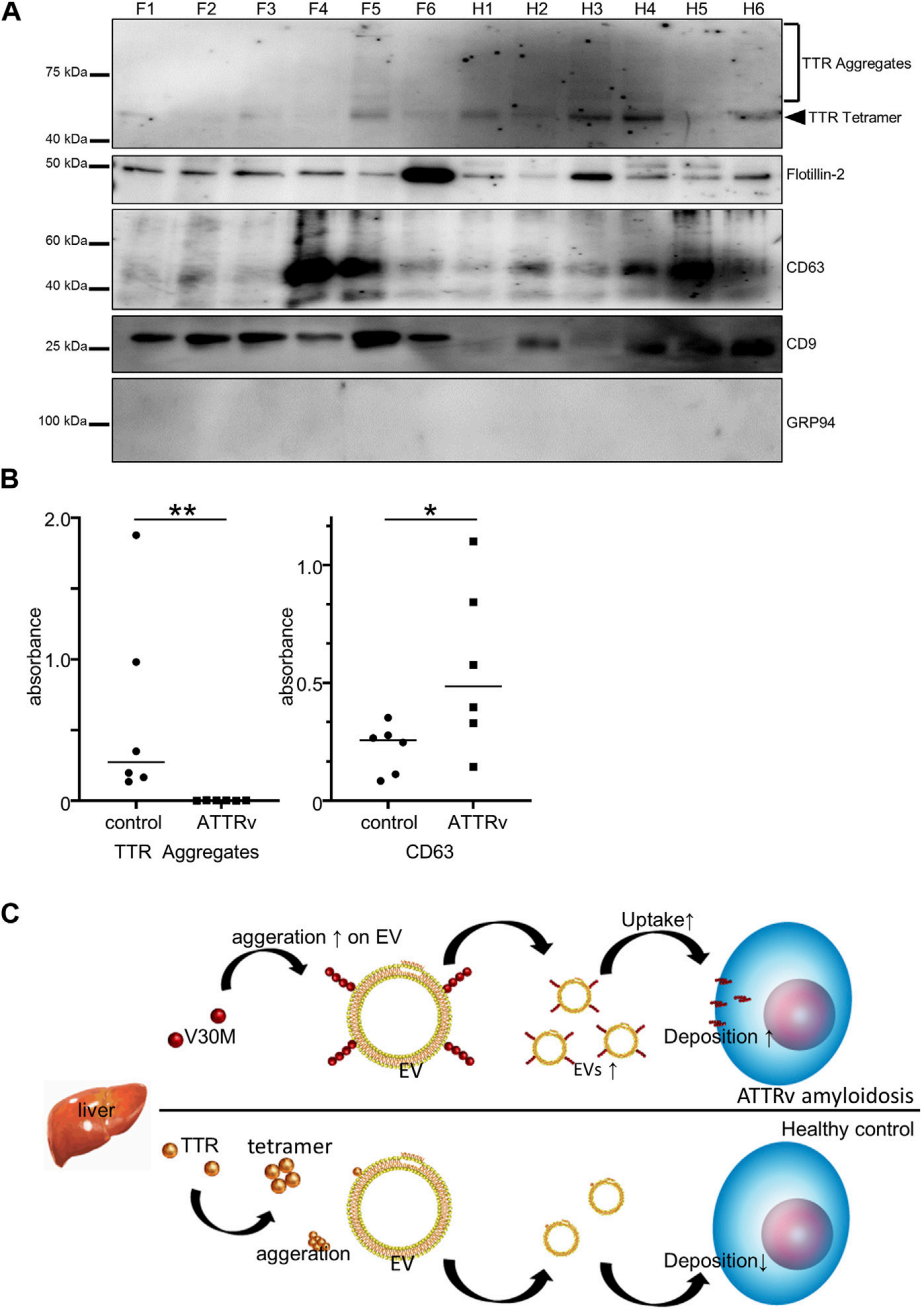
TTR in Serum-derived EVs is reduced in patients with ATTRv amyloidosis. **(A)** Western blotting analysis of S-EVs derived from patients and controls. TTR ladder bands were more obvious in the controls than in the patients. **(B)** EV ELISA using serum of controls and patients. Absorbance of TTR aggregates was significantly higher in the healthy controls than in the patients with ATTRv amyloidosis, whereas absorbance of CD63 was significantly higher in the patients than in the healthy controls. N = 6, mean ± S.E.; *, *p* < 0.05; **, *p* < 0.005, Mann–Whitney U test. **(C)** A model of EV-mediated V30M-TTR cell deposition. TTR is mainly produced by the liver and secreted into the bloodstream, but the majority of patients with ATTRv amyloidosis produce TTR with the V30M mutation, which promotes aggregation of V30M-TTR on the membranes of Serum-derived EVs and facilitates deposition on cells and other surfaces, resulting in reduced TTR in Serum-derived EVs for patients with ATTRv amyloidosis. Increasing the amount of EVs in patients with ATTRv amyloidosis could further promote this deposition.

In summary, our results showed that S-EVs contain TTR aggregates (Figure 3B) and that TTR aggregation is an important process in the pathogenesis of ATTR amyloidosis. Here, we investigated whether there is a difference in the amount of TTR aggregates and EV markers in S-EVs between patients with hereditary ATTRv amyloidosis and the healthy controls. First, we analyzed TTR aggregates by immunoblotting analysis of the EVs of patients with ATTRv amyloidosis and healthy individuals (N = 6). A higher rate of TTR tetramer and TTR aggregates was observed in the healthy controls (Figure 5A). CD9, a marker for EVs, had a high rate of low expression in healthy controls (Figure 5A). Next, we performed EV ELISA to quantify the tendency of these TTR aggregates and EV markers. Patient and control serum samples were diluted 2-fold or 16-fold, and EV ELISA was performed using a fixed volume (100 µL) of diluted serum. The absorbance was measured, and the amounts of TTR aggregates and CD63 in the serum EVs were compared. The absorbance of TTR aggregates increased to some extent in the control group, but hardly increased in the patient group (Figure 5B). The absorbance of CD63 increased in both groups but was significantly higher in the patient group than in the control group (Figure 5B). The amount of TTR aggregates on S-EVs was significantly lower in the patient group than in the healthy group, and the number of EV markers was significantly higher in the patient group than in the healthy group. Taken together, as the uptake of EVs, which promote V30M-TTR aggregation, into cells was increased by the complex of V30M-TTR and EVs (Figures 4A–C), increased EVs levels in ATTRv amyloidosis could enhance tissue deposition of TTR aggregates, which follows a decrease of TTR aggregates in the serum of patients with ATTRv amyloidosis (Figure 5C).

## Discussion

In this study, we showed that TTR present in the blood was also present in serum-derived EVs, and that V30M-TTR aggregation is promoted at the surfaces of membranes such as serum-derived EVs. V30M-TTR aggregation is particularly enhanced in the majority of patients with ATTRv amyloidosis. We also showed that monomeric TTR-V30M promoted aggregation and increased cell deposition in the presence of EVs, even under non-acidic conditions. Compared to healthy subjects, patients with ATTRv amyloidosis showed decreased TTR aggregates and increased EV markers.

In amyloidosis, the aggregation of the major causative protein is promoted on the surface of the EV membrane, which is assumed to alleviate the pathological conditions (Yuyama et al., 2012; Falker et al., 2016). TTR variant, as a major causative protein, causes TTR aggregation and deposition on specific tissues (Bezerra et al., 2020). In this study, we showed *via* atomic force microscopy that TTR aggregation is promoted by EVs and that TTR multimerizes on the surface of EVs. TTR forms a strong tetramer in serum (Kanda et al., 1974) and transports thyroxine and vitamin A through the binding region located in the central channel of the tetramer (Saponaro et al., 2020). In ATTRv amyloidosis, variants such as V30M in TTR destabilize the tetrameric structure and facilitate aggregation (Saraiva, 1995; Sekijima et al., 2005). In this study, the ThT assay and *in vitro* aggregation assay showed that V30M, a major variant of ATTRv amyloidosis, markedly enhanced TTR aggregation, even in the presence of EVs. The *in vitro* aggregation of TTR is hindered under neutral conditions and promoted under acidic conditions (Colon and Kelly, 1992). Interestingly, in the presence of EVs, TTR aggregation and cell deposition were promoted in this study, even under non-acidic conditions. The addition of purified V30M monomer alone did not cause TTR aggregation, as was observed for the addition of EVs alone as a control, suggesting that EVs did in fact induce TTR aggregation.

In Alzheimer’s disease, EVs remove Aβ, which is a toxic molecule in the brain (Pérez-González et al., 2020). EV proteins accumulate in the brain plaque of patients with Alzheimer’s disease, suggesting their strong involvement in the pathogenesis of this disease (Rajendran et al., 2006). The enhanced cellular deposition of TTR aggregates by EVs indicates that the EV-mediated tissue deposition of TTR aggregates may be involved in ATTRv amyloidosis as well as Alzheimer’s disease. In fact, the amount of EV markers in serum was significantly higher in patients with ATTRv amyloidosis than in healthy subjects. This suggests that EVs may be actively involved in ATTRv amyloidosis. However, although TTR can be transported throughout the body by the bloodstream, TTR aggregate deposits are found in specific tissues. Various membrane proteins are present on the surface of EV membranes, where they act on specific cells (Hoshino et al., 2015). Therefore, it is possible that tissue-specific deposition of TTR amyloid is mediated by the EV pathway. A detailed analysis of TTR aggregation in the EV pathway will help further elucidate the pathogenesis of ATTRv amyloidosis.

TTR has been implicated in dementia, whereby a lack of TTR can exacerbate cognitive impairment (Sousa et al., 2004). With regard to Alzheimer’s disease in amyloidosis with dementia, reduced TTR levels have been detected in both the cerebrospinal fluid and plasma of patients with Alzheimer’s disease (Han et al., 2011; Saponaro et al., 2020) and in subjects with mild cognitive impairment, becoming more pronounced as the disease progresses. Aβ, which is the major causative protein of Alzheimer’s disease, and TTR have also been analyzed molecularly. For example, TTR inhibits the primary and secondary nucleation of Aβ aggregation, restricting each the toxicity of Aβ oligomers and the proliferative ability of fibrils (Ghadami et al., 2020; Gião et al., 2020), as well as the proteolytic activity of Aβ (Saponaro et al., 2020). In other words, TTR can bind to Aβ and inhibit Aβ aggregation and toxicity, suggesting a protecting role for TTR in Alzheimer’s disease (Schwarzman et al., 1994). As Aβ promotes aggregation at the surface of EV membranes (Yuyama et al., 2012), it appears consistent with the phenomenon of TTR observed in this study. TTR is expressed in the choroid plexus in the brain (Sakai et al., 2017) in addition to the liver and Aβ is also expressed in EVs in serum across the brain blood barrier (Lim et al., 2019), suggesting that Aβ and TTR can transfer fluidly through EVs. Thus, the targeting of Aβ degradation and Aβ aggregation inhibition in the brain *via* EVs and TTR could represent a new therapeutic candidate for Alzheimer’s disease. Therefore, it is necessary to further analyze the relationship between TTR and Aβ in EVs derived from the brain, in addition to EVs derived from the liver and other blood sources.

## Data Availability

The raw data supporting the conclusion of this article will be made available by the authors, without undue reservation.
