# Supplementary material for: Extracellular Vesicles Contribute to the Metabolism of Transthyretin Amyloid in Hereditary Transthyretin Amyloidosis
Source: Front Mol Biosci. 2022 Mar 23;9:839917. doi: 10.3389/fmolb.2022.839917 (PMC8983912; doi:10.3389/fmolb.2022.839917)
Supplement: Supplementary file 1 [file DataSheet1.PDF]

Supplemental Figure 1 (Video)

High-speed atomic force microscopy analysis of S-EVs in a scanning area of 400 nm^2. S-EVs with a width of approximately 50 nm were observed.
